# Clothing for the Tropics

**Published:** 1909-05-29

**Authors:** 


### Tropical Diseases.

### CLOTHING FOR THE TROPICS.

In the *Journal of Tropical Medicine and Hygiene* for the current month there is a very interesting leader on the question of "Clothing in Tropical Countries." In this it is pointed out that suitable clothing for each individual place may generally be obtained locally, so that the amount of things to be taken out from home may be greatly reduced, and further, that much must necessarily depend on the dryness or moistness of the climate. There is no doubt that the whole question is a very large one, and a book could easily be written upon it.

So much depends on what part of the tropics the individual is going to, on what he is going to do when he arrives there, on how long he is to stay, and on a host of other things. It is certainly best, if possible, to consult someone who has recently returned from the place, because such a person must necessarily have at his finger ends the local knowledge of what is required, and can quickly give information that will save the intending traveller much time and money.

On the whole one is inclined to agree with the advice to purchase locally; but at the same time one must remember that, at least for some of the tropical African colonies, local production of clothes is impossible. On the other hand, in places as widely apart as Trinidad, Singapore, and Hong Kong, to mention only a few, the quality of the local boots, hats, underclothes, and other garments is remarkably good; but even so, one cannot expect from these the Bond Street or Regent Street cut, which to many people is indispensable. The question of the dryness or moisture of a climate and the material most suited for wearing in either is, of course, a most important item, and this must depend on the special locality, some places remaining the same all the year round, others varying considerably with the seasons. Again a great deal depends on the work of the individual. The planter or outside worker can do with less clothes than the clerk or official in the town, who has to wear linen collars and dress more like his countrymen at home. Such points as these show the difficulty of the subject generally, and how dangerous it is to dogmatise, from experiences obtained in one place, upon other countries and climates. Even in the same climate, what may suit one person may not suit another, and the best advice to give to the intending visitor to the tropics is to seek local information about the place he is going to; failing that, the big tropical outfitters in town will give a fair idea of the essential requisites, and then, when he arrives and sees for himself what his work is to be, he can supplement his stock locally or add to it from time to time.

